# Neuroinflammation and Oxidative Stress in Diabetic Neuropathy: Futuristic Strategies Based on These Targets

**DOI:** 10.1155/2014/674987

**Published:** 2014-04-30

**Authors:** Reddemma Sandireddy, Veera Ganesh Yerra, Aparna Areti, Prashanth Komirishetty, Ashutosh Kumar

**Affiliations:** Department of Pharmacology and Toxicology, National Institute of Pharmaceutical Education and Research (NIPER), Bala Nagar, Hyderabad 500037, India

## Abstract

In Diabetes, the chronic hyperglycemia and associated complications affecting peripheral nerves are one of the most commonly occurring microvascular complications with an overall prevalence of 50–60%. Among the vascular complications of diabetes, diabetic neuropathy is the most painful and disabling, fatal complication affecting the quality of life in patients. Several theories of etiologies surfaced down the lane, amongst which the oxidative stress mediated damage in neurons and surrounding glial cell has gained attention as one of the vital mechanisms in the pathogenesis of neuropathy. Mitochondria induced ROS and other oxidants are responsible for altering the balance between oxidants and innate antioxidant defence of the body. Oxidative-nitrosative stress not only activates the major pathways namely, polyol pathway flux, advanced glycation end products formation, activation of protein kinase C, and overactivity of the hexosamine pathway, but also initiates and amplifies neuroinflammation. The cross talk between oxidative stress and inflammation is due to the activation of NF-**κ**B and AP-1 and inhibition of Nrf2, peroxynitrite mediate endothelial dysfunction, altered NO levels, and macrophage migration. These all culminate in the production of proinflammatory cytokines which are responsible for nerve tissue damage and debilitating neuropathies. This review focuses on the relationship between oxidative stress and neuroinflammation in the development and progression of diabetic neuropathy.

## 1. Introduction

Diabetes mellitus is a group of metabolic disorders characterized by chronic hyperglycemia associated with symptoms like polydipsia, polyphagia, polyuria, blindness, weight loss or gain, sore heals, burning and tingling sensation, and so forth. Diabetic population of the world in 2013 was 382 million and it has been projected to rise to 592 million by the year 2035 [1]. Diabetes has become a challenging health problem affecting the global population and the prevalence is higher in developing countries. Among the top 10 countries having highest number of people with diabetes, 8 are middle-income rapidly developing countries. There will be a 42% increase in the developed countries and a 170% increase in the developing countries in diabetic cases by 2030 [2].

Diabetes is associated with both macrovascular and microvascular complications, in which the major microvascular complication is diabetic neuropathy (DN) with a prevalence of 50–60% [3]. The neuropathy progresses with decreasing nerve functionality and nerve blood perfusion which may result in malnourished nerve and leads to permanent nerve damage. The clinical manifestations of diabetic neuropathy include numbness, burning and tingling sensation, and intractable pain [4].

**Figure 1:**
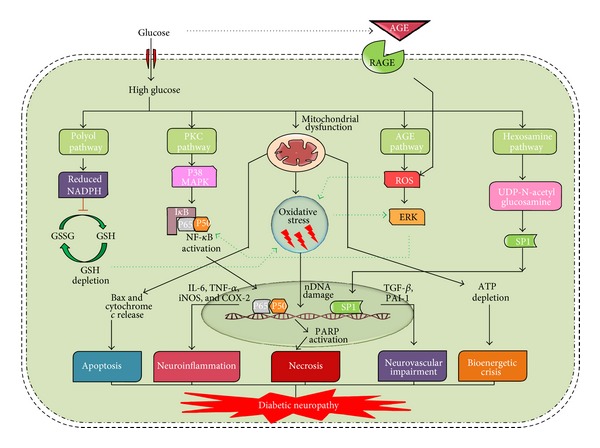
Pathophysiology of diabetic neuropathy. Hyperglycemia activates numerous metabolic pathways like polyol pathway, protein kinase c (PKC) pathway, advanced glycation end products (AGE) pathway, and hexosamine pathway. All these pathways are known to integrate through hyperglycemia mediated mitochondrial ROS production. Oxidative stress and these classical pathways in combination activate transcription factors such as nuclear factor kappa enhancer of B cells (NF-*κ*B) and speciality protein-1 (SP-1), resulting in neuroinflammation and vascular impairment. Further, these pathways combined with dysfunctional mitochondria mediated apoptosis or bioenergetic depletion can lead to neuronal damage lading to DN. Poly-ADP ribose polymerase (PARP) mediated NADH/ATP depletion can lead to neuronal dysfunction due to failure of various energy dependent processes in neurons. (ERK: extracellular related kinase, IL-6: interleukin-6, iNOS: inducible nitric oxide synthase, COX-2: cyclooxygenase-2, TGF-*β*: transforming growth factor-*β*, and PAI-1: plasminogen activator inhibitor-1.)

Although hyperglycemia is considered to be a major pathophysiological factor in the development of diabetic neuropathy, the associated mechanisms are not fully understood yet. Some of the major pathways like polyol pathway [5], advanced glycation end products [6], hexosamine flux [7], mitogen-activated protein kinases [8], altered activity of Na^+^/K^+^-ATPase [9], poly-ADP ribose polymerase (PARP) over activation [10], and cyclooxygenase-2 (COX-2) activation [11] have been reported to play a crucial role in development and progression of diabetic neuropathy (Figure 1). Nerve cells are prone to hyperglycemic injury as the neuronal glucose uptake is based on external glucose concentration which is 4-5-fold higher in diabetic subjects. It has been noted in experimental diabetes that the levels of neurotrophic support, including nerve growth factor and insulin like growth factor are reduced [12], which also contribute to malnourishment of nerves. All these pathways form a common platform with end result as neuronal dysfunction and nerve damage and this translates in the development of various clinical deficits seen in patients suffering from diabetic neuropathy.

Treatment of painful diabetic neuropathy presents a great challenge to clinicians due to poor diagnostic criteria and the limited treatment options available. Currently, drugs in clinical use for diabetic neuropathic pain include tricyclic antidepressants, selective serotonin and noradrenaline reuptake inhibitors, anticonvulsants, and opioids [13]. Although there is a range of pharmacological agents available for treating the pain associated with diabetic neuropathy, only duloxetine and pregabalin are approved by US Food and Drug Administration (US FDA) for the treatment of diabetic neuropathic pain. However, as single agents, they are limited by incomplete efficacy, high cost, and dose limiting adverse effects [14–16].

In spite of the voluminous research done in the field of diabetic neuropathy, clear understanding of the pathophysiology and the interwoven mechanisms are still lacking. There is a need to investigate futuristic, combinational, and other nonpharmacological approaches for alleviating DN and associated neuronal deficits.

## 2. Classical Pathways of Hyperglycemic Vascular Damage

Diabetic neuropathy is a syndrome which can affect both the somatic and autonomic divisions of the peripheral nervous system. In severe diabetic conditions, longer nerve fibres show an earlier loss of nerve conduction velocity with loss of their nerve terminals. The damaged nerve terminals are the reason for tingling and loss of sensation and reflexes are often first observed in the feet and then they ascend to affect other areas [4]. One of the major causes for all these complications is reactive oxygen species (ROS) produced from processes initiated and amplified under chronic hyperglycemic conditions. Further, classical hyperglycemic pathways like polyol pathway, protein kinase C (PKC) pathway, formation of advanced glycation end products (AGE pathway), and hexosamine pathway activation leads to aggravation of oxidative damage leading to vascular complications [17]. In polyol pathway, aldose reductase enzyme converts glucose into sorbitol, which is then oxidized into fructose by sorbitol dehydrogenase (SDH) with NAD^+^ as a cofactor. In case of hyperglycemic conditions, increase in oxidative stress directly results from the accumulation of sorbitol and indirectly through consumption of NADPH, a cofactor for the regeneration of reduced glutathione (GSH) [18]. Increased flux through polyol pathway can decrease (Na^+^/K^+^) ATPase activity and studies suggested that decreased activity of this enzyme may activate PKC pathway [19]. Activation of PKC increases cytosolic phospholipase A2 activity and produces arachidonate and prostaglandin E_2_ (PGE_2_) which effectively inhibits cellular (Na^+^/K^+^) ATPase [20]. Persistent and excessive activation of several PKC isoforms initiates tissue injury by diabetes-induced ROS [21], which leads to enhanced de novo synthesis of DAG from glucose via triose phosphate. Increased levels of triose phosphate concentrations can increase the formation of both methylglyoxal, a precursor of AGEs, and diacylglycerol (DAG), an activator of PKC [22]. Evidence suggests that the enhanced activity of PKC isoforms results in activation of various signalling mechanisms like mitogen-activated protein kinases (MAPK), nuclear factor kappa light chain enhancer of B cells (NF-*κ*B), and thus leads to initiation of inflammation as depicted in Figure 1. Overactivation of PKC has been also implicated in the decreased nitric oxide (NO) production in smooth muscle cells and increased expression of fibrinolytic factor, plasminogen activator inhibitor (PAI-1), tumor growth factor-*β* (TGF-*β*), and NF-*κ*B activation in cultured endothelial cells and in case of vascular smooth muscle cells [23]. AGE pathway activation results in production of many advanced glycation end products, which act on specific receptors like receptor for advance glycation end products (RAGE) present on monocytes and endothelial cells to increase the production of cytokines and adhesion molecules and also causes the alteration of protein structure. AGEs have been shown to have an effect on matrix metalloproteinases, which might damage nerve fibres [24]. AGE receptor ligation can activate transcription of pleiotropic factor NF-*κ*B and thus enhances the production of various proinflammatory mediators (Figure 1) [25]. Hyperglycemia and insulin resistance induced excess fatty acid oxidation also appears to be reason for pathogenesis of diabetic complications [26]. In hexosamine pathway, fructose-6-phosphate converts to glucosamine-6-phosphate by glutamine fructose 6-phosphate amidotransferase (GFAT). Glucosamine-6-phosphate then converts into UDP-N acetyl glucosamine with the help of specific O-GlcNAc transferases. Evidence suggest that inhibition of GFAT blocks hyperglycemia-induced transcription of both TGF-*α* and TGF-*β*1 [27]. The mechanism behind the hyperglycemia-induced expression of genes such as PAI-1, tumor growth factor-*α* (TGF-*α*), and TGF-*β*1 is not clear. However, it has been observed that hyperglycemia causes a fourfold increase in O-GlcN acylation of the transcription factor specificity protein 1 (Sp1), which mediates hyperglycemia-induced activation of the plasminogen activator inhibitor-1 (PAI-1) promoter in vascular smooth muscle cells and of TGF-*β*1 and PAI-1 in arterial endothelial cells (Figure 1) [28]. Activation of PAI-1, TGF-*α*, and TGF-*β*1 causes accumulation of extracellular matrix which may lead to neuroinflammation associated with diabetic neuropathy [29].

## 3. Oxidative Stress and Mitotoxicity: Role in Neuronal Dysfunction

Hyperglycemia induces activation of classical pathways like AGE, PKC, hexosamine, and polyol pathways to mediate cellular damage [17]. However, the hyperglycemic cell injury is the result of cumulative occurrence of this cascade of pathways discussed in the previous section [22].

Generation of superoxide from mitochondrial electron transport chain is known to contribute towards hyperglycemia initiated various etiological pathways. Hyperglycemia enhances the reducing equivalents to electron transport chain (ETC) and the electrochemical potential across the inner mitochondrial membrane and hence increases superoxide production [22]. Superoxide inhibits glyceraldehyde phosphate dehydrogenase (GAPDH) either directly or indirectly through PARP mediated NADH^+^ depletion [30, 31]. Inhibition of GAPDH by ROS leads to accumulation of glycolytic intermediates upstream of this enzyme and redirected to initiate cellular pathways like AGE formation. Once the AGEs are formed, they bind to RAGE and activate many other crucial pathways like NF-*κ*B and PARP. PKC pathway is activated through dihydroxy acetone phosphate mediated diacylglycerol (DAG) activation. Hexosamine pathway which is activated through enhanced flux of fructose-6-phosphate and polyol pathway by elevated glucose levels [17]. This, in turn, leads to osmotic stress in the cells which further takes the cell towards necrotic cell death. Enhanced activity of Mn-SOD, a mitochondrial form of superoxide dismutase (SOD) or overexpression of uncoupling proteins (UCP-1) in experimental diabetic animals, prevents the development of vascular complications in the animals and also reduced oxidative stress mediated neuronal damage [31, 32]. The mechanism for this neuroprotective effect can be the reduction of mitochondrial ROS generation and the clearance of the notorious ROS from the cells.

In addition to the above theory, mitochondrial abnormalities and mitochondria associated oxidative stress stands at a central position in the pathogenesis of diabetic neuropathy [33]. It has been noticed that defects in functioning of ETC chain components compromises ATP production and enhances the generation of free radicals. The free radicals generated causes damage to mitochondrial DNA (mt DNA) and nuclear DNA (n DNA) which in turn aggravates mitochondrial damage [34]. This vicious cycle developed inside mitochondria produces intense oxidative stress and drives the cell towards apoptotic/necrotic death [35]. It is an established fact that diabetes is known to affect the respiratory capacity of ETC functional complexes and thus alters ATP production (Figure 1). Mainly complex I and complex III are known to be affected, which turn out to be electron leakage centres and thus inflates ROS production [34].

In addition to disturbed mitochondrial functionality, its dynamics (size, shape, and number) is also known to be affected in diabetic neuropathy [36]. Changes in mitochondrial morphology, movement characteristics can affect the transfer in axons which can lead to various sensorimotor changes. The glove and stocking pattern of thermal sensitivity is due to impairment in the anterograde axonal transport in sensory neurons [37]. Dysfunctional mitochondria can also mediate cell death through execution of apoptotic pathways, by releasing pro apoptotic factors from mitochondria into the cytosol [35]. Various experimental observations point towards the critical role of mitotoxicity in the pathophysiology of diabetic neuropathy.

## 4. Neuroinflammation and Role in Peripheral Nerve Damage

**Figure 2:**
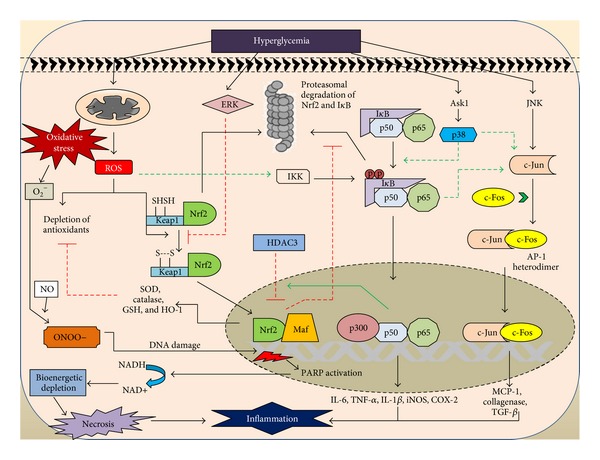
Crosstalk between oxidative stress and inflammation. Hyperglycemia mediated oxidative stress and inflammatory pathways are known to interact with each other at various levels. ROS activates nuclear factor (erythroid-1) related factor (Nrf2) by directly oxidising the thiol residues on kelch-like ECF associated protein (Keap-1). Nrf2 then migrates into the nucleus to activate antioxidant response elements (ARE) of genome. However, this Nrf2 activation by hyperglycemia is inhibited through extracellular related kinase activation (ERK). ROS also activates inhibitory kappa B kinase (IKK), which then phosphorylates the inhibitory kappa B protein (I*κ*B); the latter combines with cytosolic NF-*κ*B complex ant thus preventing its transcription. Phosphorylation of I*κ*B labels it for ubiquitination and proteasomal degradation and, hence, releases NF-*κ*B complex to enter into nucleus, which then expresses several proinflammatory mediators. Similarly, oxidative stress mediated c-JUN N terminal kinases (JNK) activation mediates the c-JUN component of activator protein-1 (AP-1) activation, which then combines with c-FOS subunit. The resulting AP-1 heterodimer binds with genome and increases production of various vascular inflammatory mediators. Oxidative stress mediated PARP activation also leads to inflammation through necrotic cell death. Nrf2 inhibits I*κ*B degradation and thus prevents NF-*κ*B mediated inflammation. NF-*κ*B also prevents the Nrf2 signalling through histone deacetylases (HDAC3) recruitment (ASK-1-apoptosis signalling related kinase-1 and MCP-1-monocyte chemoattractant protein-1).

Diabetic peripheral neuropathy is characterized by debilitating pain and sensory loss which leads to diminished quality of life. Persistent hyperglycemia is believed to be underpinning for the neuroinflammation and nerve damage leading to the neuropathic pain. All the characterized classical pathways like polyol pathway, PKC pathway, MAPK pathway, and increased production of AGEs could directly or indirectly initiate and progress the production of inflammatory mediators [13]. Especially the accumulation of AGE products of proteins and lipids stimulate the generation of inflammatory mediators and activation of transcription factor NF-*κ*B, a potent inducer of inflammatory processes [38]. These AGEs act on various receptors present on microglia and macrophages stimulate production of cytokines like IL-1, IL-6, IL-17, TNF-*α*, chemo attractant protein-1, C-reactive protein and chemokines like CCL-2, CXC, and so forth (Figure 1) [39, 40]. Activation of RAGE can induce inflammatory cascade through the activation of NF-*κ*B pathway. NF-*κ*B is a transcription factor that upregulates the gene expression of proinflammatory cytokines and also is responsible for the induction of neuronal apoptosis. Activation of NF-*κ*B also suppresses the expression of antioxidant genes by downregulating Nrf-2 pathway and thus indirectly weakening the innate antioxidant defense (Figure 2) [41]. Persistent hyperglycemia induced inflammation also affects the structural features of neuron as the glycosylation of myelin protein alters its antigenicity causing infiltration of monocytes, macrophages, neutrophils from the blood circulation, and activation of glial cells of the nervous system [24, 42]. These immune cells in turn secrete inflammatory cytokines which further damages myelin sheath and increases nerve excitability, thus leading to edema and neuroinflammation. The stimulated monocytes and immune cells have a vicious positive feedback loop for increasing the production of inflammatory mediators thus potentiating nerve catastrophe. The cytokines like IL-1, IL-6, and IL-17 can sensitize the peripheral receptors causing neuropathic pain [43]. Additionally, neuroinflammation leads to nerve damage due to apoptosis induced by MAPK signalling [44]. TNF-*α* also promotes the expression of cell adhesion molecules which are capable of decreasing the blood perfusion rate and thus decreases neurotrophic support [42]. The released chemokines have been shown to produce hyperalgesia through the activation of chemokines receptors present on the nerves. Hypoxia and ischemia created in diabetes also aggravate the neuroinflammation through the induction of inducible nitric oxide synthase (iNOS), which releases NO, a physiological mediator of inflammation [45]. In large, activation of inflammatory cascade, proinflammatory cytokine upregulation, and neuroimmune communication pathways plays vital role in structural and functional damage of the peripheral nerves leading to the diabetic peripheral neuropathy.

## 5. Cross Talk between Inflammation and Oxidative Stress

Hyperglycemic condition is known to activate both oxidative stress and inflammatory pathways. The interaction of these two pathways complicates the hyperglycemia mediated neuronal damage. The oxidative stress induced ROS and various constitutional inflammatory pathways are known to interact at multiple levels to produce plethora of pathophysiological outcomes (Figure 2) [46].

Hyperglycemia is known to increase metabolic flux through mitochondrial electron transport chain, leading to inefficient electron transfer through redox centres and hence generating superoxide anion [22]. Excessive superoxide generation leads to production of other ROS such as H_2_O_2_, OH^−^. Superoxide can also combine with nitric oxide (NO) to produce peroxynitrite (ONOO^−^), a potent reactant which causes nitration of several important proteins and leads to structural and functional damage [47]. Peroxynitrite mediated DNA damage leads to activation of PARP, a nuclear enzyme which causes transfer of poly-ADP ribose units to DNA by utilising NADH energy pool. Depletion of NADH leads to bioenergetic crisis and thus drives the cell towards necrosis [48]. Necrotic cell death is known to release cellular debris, which further drives the inflammatory cells to the damaged spot and hence activates a local inflammatory episode (Figure 2).

Hyperglycemia mediated oxidative stress also activates other cellular pathways like Nrf2 and NF-*κ*B. Activation of Nrf2 pathway enhances the production of several antioxidant and cytoprotective enzymes through transcriptional facilitation of antioxidant response element (ARE) of genome. These enzymes include SOD, GSH, HO-1, and glutathione s-transferase (GST) [49]. Activation of this Nrf2 pathway stands as one of the cellular homeostatic mechanisms to protect cells from enhanced oxidative stress. However, persistent Nrf2 activation is subdued through hyperglycemia mediated ERK activation, and hence redox homeostasis is failed in diabetic state as depicted in Figure 2 [50].

Oxidative stress mediated inflammation is known to execute NF-*κ*B, activator protein-1 (AP-1), and MAPK pathways. ROS are known to activate inhibitory kappa –B kinase (IKK), which causes phosphorylation of I*κ*B, labeling it for ubiquitination mediated proteasomal cell death [51]. Release of free NF-*κ*B heterodimer from I*κ*B allows it to cross nuclear membrane and bind with kappa region of genome. Transcriptional facilitation of this kappa region of genome enhances the production of inflammatory cytokines such as TNF-*α*, IL-6, COX-2, and iNOS as shown in Figure 2 [52]. Upstream of this pathway, NF-*κ*B activation at cytosolic level is also facilitated by p38 MAPK. This p38 MAPK is known to be activated directly through hyperglycemia mediated apoptosis signal regulating kinase1 (ASK1) or indirectly through oxidative stress. Oxidative stress is also known to activate stress activated protein kinases, that is, c-Jun N-terminal kinases (JNK), which further activates JUN subunit of AP-1 and hence facilitate AP-1 mediated collagenase, TGF-1*β*, and other cytokines production (Figure 2) [53]. Although AP-1 involvement in the pathogenesis of DN needs to be explored, its activation can produce a local sequela of vascular inflammation and thus support the rationale for its participation in neuroinflammation.

Among the above mentioned pathways, the crosstalk between Nrf2 and NF-*κ*B is critical both physiologically and pharmacologically [41]. Activation of Nrf2 pathway is known to inhibit NF-*κ*B activation through reduced ROS mediated IKK activation and by inhibiting the degradation of I*κ*B. Further activation of NF-*κ*B competes with Nrf2 for binding to antioxidant response element (ARE), either directly or indirectly through recruiting histone deacetylase 3 (HDAC3) to the ARE region (represented in Figure 2) [54]. Interaction between these two pathways maintains the cellular homeostasis. However, diseases associated with excessive oxidative stress generation can cause imbalance in Nrf2-NF-*κ*B axis and thus produce damaging consequences [41].

There is much scientific evidence supporting the involvement of inflammatory pathways in direct peripheral nerve damage and neuroinflammation. However, a growing body of researchers suggests that neuroglial cells act as connecting link between oxidative stress and neuroinflammation [55]. According to this theory, oxidative damage to glia produces excessive proinflammatory cytokines, which in turn acts on membrane receptors of neuronal cells and thus activates inflammatory pathways, causing neuroinflammation [56]. There is also evidence supporting the role of vascular inflammation in the pathogenesis of diabetic neuropathy. Accumulation of all these evidence suggests that neuroinflammation is not the sole episode underlying peripheral nerve damage but it is accompanied by inflammation and oxidative, nitrosative stress in the vasa nervorum and neuroglial cells.

## 6. Futuristic Strategies for Diabetic Neuropathy

Identification of pathomechanisms underlying disease pathogenesis is important to not only devise new treatment strategies but also be useful in discovering new disease biomarkers. Biomarker identification can be useful to identify the extent of disease progression and thus can amplify the scope of better drug targeting. Currently available diagnostic methods for DN include assessment of vibration perception threshold (VPT) and calculation of neuropathy disability score (NDS) based on ankle reflexes and perception changes to variety of stimuli [57]. Newer techniques with minimal invasion or noninvasive operation include corneal confocal microscopy (CCM) and skin biopsy techniques. CCM allows the identification of corneal nerve fibre length and nerve density and thus can be used as diagnostic aid to quantify peripheral neuropathy [58]. Skin biopsy and consequent immunohistochemistry allow the quantification of the number of nerve fibres per unit area [59]. However, these diagnostic procedures can be combined with examination of biochemical changes to accurately monitor the disease progression and response to treatment.

Based on the compelling evidence put forth by many research groups, it is being clear that oxidative stress mediated neurodegeneration and the accompanied inflammatory reactions play a prominent role in the pathogenesis of diabetic neuropathy [13]. Modulation of these pathways by pharmacological agents can prevent the functional and pathophysiological disturbances associated with peripheral neuropathy and can accelerate the discovery of new treatment strategies for diabetic neuropathy. Some of the important categories of drugs which have potential to affect the oxidative stress and inflammatory pathways in relevance to peripheral neuropathy will be discussed in the following section.

Oxidative stress is also known to enhance the endoplasmic stress through accumulation of misfolded proteins. Recently, the role of ER stress in the pathogenesis of diabetic neuropathy has been well observed. ER plays an important role in the proper folding and processing of proteins. Oxidative damage to the ER causes dysfunctional protein processing system and enhances the accumulation of nonfunctional proteins [60]. Chaperons are the ER proteins which help in processing newly synthesized proteins. Administration of chemical chaperons such as trimethylamine oxide and 4-phenyl butyric acid was found to inhibit the diabetes associated oxidative stress in spinal cord and dorsal horn, reduce intraepidermal nerve fibre loss, and ameliorate peripheral nerve damage and thus it can be used as a therapeutic strategy for diabetic neuropathy [61].

Nitrosative stress is also considered to be equally contributing in the pathogenesis of diabetic neuropathy as similar to oxidative stress [62]. Primarily, peroxynitrite is the toxicant of this pathway which causes biomolecular damage and PARP activation. PARP activation further depletes cellular energy pool and causes necrotic cell death [47]. Use of peroxynitrite decomposition catalysts and PARP inhibitors prevent the neuronal damage associated with diabetic neuropathy. Several trails done with these agents could alleviate the biochemical and functional impairment produced due to diabetes in sciatic nerves and dorsal root ganglion (DRG) neurons [63, 64].

Due to massive involvement of oxidative stress in the pathogenesis of diabetic neuropathy, several antioxidants have been tried in patients with diabetic neuropathy. Alpha-lipoic acid, vitamin E, and acetyl-L-carnitine were studied clinically in several controlled prospective clinical trials [65–67]. Among them alpha-lipoic acid was shown to relieve sensory and functional deficits of DN and has been approved by FDA for therapeutic use [68].

Impaired synthesis of vasoactive prostanoids and associated endothelial dysfunction is one of the pathological factors contributing to DN, which is initiated by both oxidative stress and inflammation. Diabetic neuropathy is associated with compromised blood flow which results in lack of endonutritive blood supply to neurons which may be directly or indirectly related to oxidative stress directed endothelial damage in vasa nervorum [69]. Several vasodilators like angiotensin receptor antagonists, endothelin antagonists, phosphodiesterase inhibitors, calcium channel antagonists, nitro vasodilators, and prostanoid analogues have been tested in animal models of diabetes and among them angiotensin II receptor antagonists (e.g., ZD7155) and ET_B_ receptor antagonists (e.g., BMS 182874) found to alleviate neurovascular deficits in STZ induced diabetes model [70, 71]. However, their clinical success needs to be explored to evaluate their therapeutic use in diabetic neuropathy. Enhanced oxygen delivery to peripheral nerves result in increased nerve regeneration through counteraction of ischemic, hypoxic, inflammatory, and necrotic episodes associated with diabetic neuropathy [72].

Neuroinflammation occurs when there is a persistent release of proinflammatory mediators and the pathways are activated through corresponding cytokines in neuronal cells. The proinflammatory mediators include TNF-*α*, IL-6, IL-1*β*, COX-2, and iNOS as well as several chemokines [44]. Antibodies or chemical agents against these cytokines and chemokines could alleviate the proinflammatory episode associated with diabetic neuropathy [42, 73]. These agents are known to inhibit the consequences of inflammatory changes associated with neuroglial activation. Transcriptional modulators of NF-*κ*B and MAPK can provide a two-tier targeting approach for the prevention of neuroinflammatory changes in DN.

NF-*κ*B and Nrf2 pathways are two important pathways mediating cellular homeostasis through controlling oxidative stress and inflammation. As discussed in the above section, deregulation in the balance of Nrf2–NF-*κ*B axis may lead to several pathophysiological consequences and hence modulators of these pathways can be used to prevent such results [41]. NF-*κ*B pathway involvement in the pathogenesis of diabetic neuropathy was well documented. Several natural inhibitors of NF-*κ*B like curcumin, resveratrol, and melatonin and small molecule modulators of this pathway (BAY 117082, JSH23) were used in experimental diabetic animals [41, 74–76]. These drugs were shown to be promising by modifying the sensorimotor functional and proteomic changes associated with neuropathy. The use of NF-*κ*B inhibitors can prevent the AGE mediated proinflammatory cytokine production and thus halts the events associated with neuroinflammation. Similarly, it is being observed that overt oxidative stress in neuronal cells is a pivotal pathogenetic mechanism in nerve damage, which can be prevented by enhancing Nrf2 mediated ARE gene expression. Nrf2 enhances the production of antioxidant and cytoprotective enzymes which counteract oxidative stress. Several pharmacological antioxidants have been known to enhance Nrf2 mediated antioxidant expression in experimental models of diabetic neuropathy and found to improve behavioural, functional, and biochemical characteristics associated with diabetes [77, 78]. Rather than individually targeting Nrf2 and NF-*κ*B, pharmacological modulators of both transcription factors can produce a better therapeutic response by simultaneously enhancing Nrf2 and inhibiting NF-*κ*B [41].

Since mitochondria are the primary source of super oxide, mitochondria targeted antioxidants can reduce the corresponding oxidative damage. Several antioxidants like *α*-lipoic acid and N-acetyl cysteine are shown to have therapeutic efficacy in animal and human diabetic neuropathy [68, 79]. However, targeting the antioxidant molecules directly to the mitochondria not only reduces oxidative stress but also inhibits the other pathophysiological pathways associated with mitochondrial dysfunction. Antioxidant molecules can be effectively conjugated to lipophilic cationic molecules like triphenyl phosphonium (TPP+) and hence accumulate in the mitochondria based on the large negative potential inside the mitochondrial membrane [80]. Mito Q, Mito vitamin E, Mito PBN, and so forth are the examples of drugs that were delivered to mitochondria in various experimental setups. Other strategies like Szeto-schiller (SS) peptides can also be conjugated to antioxidants to attain maximum concentration of drug inside the mitochondrial matrix. These peptides comprise four alternative aromatic/basic amino acid back bones and direct the targeted antioxidants to inner mitochondrial membrane. These SS peptides scavenge hydrogen peroxide and peroxynitrite radicals and are known to inhibit lipid peroxidation reactions effectively [81]. These drugs have shown beneficial effect in the preclinical models of diabetic neuropathy and need to be further assessed clinically [82]. Along with mitochondrial antioxidants, drugs which can increase the mitochondrial function can alleviate bioenergetic crisis and ETC dysfunction associated with DN. One such example of drugs includes PGC-1*α* modulators, which can rescue the mitochondrial dysfunction by enhancing the production of mitochondrial enzymes and mtDNA transcription through nuclear respiratory factor 1 (Nrf1) activation [83]. PGC-1*α* activation is also known to reduce the oxidative damage through enhanced Nrf2 activation [84].

## 7. Summary

Oxidative stress and neuroinflammation are identified to be pivotal pathophysiological triggers in various diabetes associated microvascular complications including diabetic neuropathy. The use of drugs targeting oxidative stress-inflammatory pathways was found to improve the sensorimotor and functional deficits associated with diabetic neuropathy. But their clinical success remained inferior due to complexity in cellular redox signalling pathways and its further interaction with cellular kinome, genome, and epigenome. Since, redox imbalance produced in one pathway can elicit another pathway, combinational use of several strategies mentioned above could produce more beneficial effects than monotherapy. Mitochondrial dysfunction is known to initiate the hyperglycemic cellular injury; the use of drugs targeting mitochondria will find greater attention in the near future for the treatment of diabetic neuropathy. Still, a lot of work is warranted to further elucidate the cross talk of oxidative stress, mitochondrial dysfunction, and inflammation in the pathophysiology of diabetic neuropathy.
